# The spillover effects of formal social support on vulnerability to poverty among rural older adult households: a risk ‘shock-response’ perspective

**DOI:** 10.3389/fpubh.2025.1710527

**Published:** 2026-01-20

**Authors:** Cui Fu, Zhiren Tan, Jun He

**Affiliations:** 1 School of Economics and Management, Nanjing Agricultural University, Nanjing, Jiangsu, China; 2 School of Economics, Beijing Institute of Technology, Beijing, China

**Keywords:** formal social support, informal social support, rural older adult households, spillover effects, vulnerability to poverty

## Abstract

**Background:**

Mitigating vulnerability to poverty among rural older adult households remains a critical challenge for developing countries striving to achieve poverty reduction goals. While the role of formal social support in alleviating rural poverty is recognized, the specific mechanisms through which it mitigates vulnerability among rural older adult households have not been sufficiently explored.

**Methods:**

Using longitudinal data from the 2018–2022 waves of the China Family Panel Studies (CFPS), this study examines how formal social support affects the vulnerability to poverty among rural older adult households from a risk “shock-response” perspective. We categorize formal social support into preventive social support (PSS), risk mitigation social support (MSS), and safety net social support (SSS), while further investigating its spillover effects and regional heterogeneity.

**Results:**

(1) formal social support significantly reduces vulnerability to poverty among rural older adult households; (2) a positive spillover effect exists, wherein formal social support fosters non-economic support provided by adult children and neighbors; and (3) this spillover effect exhibits significant regional heterogeneity, being notably weaker in Northeast China compared to other regions.

**Conclusion:**

These findings underscore the criticality of understanding formal support mechanisms to optimize social support systems and refine poverty reduction governance. Particularly in the context of population mobility, these insights are vital for alleviating vulnerability to poverty among rural older adult households in China and addressing broader challenges of rural poverty in developing countries.

## 1 Introduction

Poverty remains an enduring global challenge, prompting the United Nations to prioritize *No Poverty* as the first of its core Sustainable Development Goals (1). Despite significant strides in global poverty reduction over the past three decades, poverty persists as a critical issue, particularly within the rural regions of developing countries. The United Nations reports that nearly four-fifths of the world’s poor reside in rural areas. This distribution not only reflects the regional characteristics of poverty but also underscores the persistent challenges developing nations face in promoting balanced urban–rural development and reducing inequality. More recently, the global poverty landscape has been exacerbated by the COVID-19 pandemic. The pandemic’s direct economic shocks and indirect social impacts have significantly heightened the vulnerability of populations previously lifted out of poverty, increasing the risk of re-impoverishment. According to the *UN Working Paper 2023*, the pandemic pushed nearly 90 million additional people into extreme poverty, with projections suggesting that approximately 7% of the global population will remain in extreme poverty by 2030 (2). Consequently, poverty alleviation remains a paramount challenge for many countries.

While countries have made progress toward the global goal of eradicating all forms of poverty, health-related poverty, particularly among the older adults, remains a severe issue. Moreover, the older adults in developing countries are disproportionately vulnerable to poverty (3). The World Health Organization (WHO) reports that one-sixth of the global population will be aged 60 or older by 2030.^1^ This demographic shift, compounded by the profound impacts of urbanization and population mobility on the lifestyles of the older adults, has established aging as a global challenge. Although population aging initially emerged in high-income nations, the trend is now accelerating rapidly in low- and middle-income countries. In China, the issue is particularly acute, driven by rapid improvements in living standards and the historical implementation of the Family Planning Policy. Data from the National Bureau of Statistics (NBS) indicate that 21.1% of the Chinese population was aged over 60 by the end of 2023.^2^ Influenced by industrialization and urbanization, the dynamics of poverty and its associated risks are especially pronounced in rural areas. Research indicates that old age is associated with the highest incidence of poverty across the life course (4). Due to declining physical function, weakened labor capacity, and the erosion of traditional household support functions, the income of the rural older adults is often drastically reduced or entirely lost (5–7). Consequently, limited income sources combined with resource constraints render the rural older adults highly susceptible to falling into poverty. Although China has achieved the UN’s 2030 poverty reduction goal a decade ahead of schedule, the rural older adults remain a critical priority in poverty governance due to their severe livelihood vulnerabilities in the context of rapid aging.

Much of the existing research on older adult poverty has focused on *ex-post*, static analyses (8). Indeed, dynamic analysis from an *ex-ante* perspective is critical for understanding and preventing poverty in older adult households, as it would effectively mitigates the occurrence of poverty at its source. In 2001, the World Bank introduced the concept of vulnerability to poverty by linking poverty and vulnerability (9). This concept expands the conceptual scope of poverty and provides a forward-looking measurement tool for the pre-identification of rural older adult households at risk of falling into poverty. Previous studies have conceptualized vulnerability as arising from the interplay between risk shocks and a lack of risk response capacity (10). Therefore, within the risk ‘shock-response’ framework, the vulnerability to poverty among rural older adult households can be analyzed from multidimensional perspective. On the one hand, rural older adult households are increasingly vulnerable to health risk shocks due to physiological decline (11). On the other hand, they often possess a diminished capacity to respond to risks due to limited resource endowments and inadequate social security. The interaction of these factors renders rural older adult households particularly vulnerable to poverty.

Addressing the vulnerability to poverty among rural older adult households necessitates mitigating risk shocks and enhancing risk response capacity. As individuals age, the continuous depreciation of health human capital not only constrains social engagement and labor supply among the older adults but also imposes a direct financial burden through escalating chronic disease and long-term care expenditures. Data indicate that approximately 70% of an individual’s lifetime medical expenditures occur after the age of 65.^3^ These statistics underscore the substantial financial burden placed on the older adults and highlight the profound impact of health shocks on the economic stability of rural older adult households. This burden is disproportionately heavier in rural areas, exacerbated by the scarcity of medical resources and structural barriers to service access (12). International research further indicates that in developing contexts, poor health is inextricably linked to vulnerability and economic insecurity. The older adults become significantly more susceptible to poverty and the accumulation of disadvantage without adequate social security (13, 14). The COVID-19 pandemic has further exacerbated these challenges (15). Research indicates that loneliness and social isolation among the older adults significantly increased during the pandemic, conditions known to precipitate adverse mental health issues such as depression and anxiety. Complementing these findings, empirical studies from Europe demonstrate that the older adults experienced deteriorating health outcomes during the crisis, leading to a heightened reliance on both informal care networks and social services (16).

In addition to the financial burdens related to health problems, rural older adult households also face weakened household support systems. In China, the traditional culture of filial piety, specifically the concept of raising sons for old-age support, has long been a vital safety net for the rural older adult households (6). However, rapid aging and the migration of the rural labor force have brought new challenges. Data from the 2020 Chinese National Census show that family size is shrinking, with the average household now having fewer than three members.^4^ This demographic change leads to less informal social support for rural older adult households (17). As the young workforce moves to urban areas, the traditional support from adult children is declining, which further weakens the older adult care function of families. Furthermore, compared to urban residents, the rural older adults have limited access to formal social support such as health insurance and pensions (18). This lack of resources makes them more vulnerable to various risks.

The *UN’s World Social Report 2023* calls for supporting the aging global population, emphasizing the need to build sustainable social protection systems.^5^ This provides a clear policy reference for countries on how to use formal systems to provide a safety net and empowerment against the backdrop of weakening family support and population aging. However, given the constraints of limited resources for formal social support, enhancing risk response capacity of rural older adult households through optimizing the interaction between formal and informal social support has become a crucial issue to prevent them from falling into poverty. Existing research is still insufficient in exploring how formal and informal social support systems can work synergistically, particularly in the context of the labor mobility. We focus on addressing this research gap by asking: how does formal social support, once embedded at the household and community levels, work synergistically with informal social support to reduce the vulnerability to poverty among rural older adult households? By answering this question, we can enhance our understanding of the sources of vulnerability to poverty among rural older adult households and lay a theoretical foundation for developing more comprehensive and effective anti-poverty policies.

We propose a theoretical framework that incorporates the context of labor migration. We believe that formal social support serves a critical role in risk response by providing essential security. Meanwhile, formal social support also generates spillover effects by reinforcing informal social support from neighbors through fostering generalized trust. It should be noted that informal social support also encompasses economic and non-economic support from adult children. However, we posit that the provision of economic support from adult children may transition toward life caregiving and emotional support when formal social support is available, reflecting the spillover effects of formal social support at the household level. Furthermore, in labor outflow areas, rural older adult households lack the informal social support due to the migration of younger generations. Formal social support is often more critical in alleviating vulnerability to poverty in such contexts. To empirically validate these hypotheses, we use data from the waves of 2018–2022 CFPS to estimate the impact of formal social support on three dimensions: (i) the vulnerability to poverty among rural older adult households, (ii) the informal social support, and (iii) the regional heterogeneity of these effects within labor migration contexts.

Our paper makes several contributions to the literature. Firstly, it extends the welfare analysis of the rural older adults from an ex-post perspective to a dynamic, focusing on vulnerability to poverty from risk shock-response perspective. Secondly, the findings on spillover effects of formal social support enrich the social policy and gerontology literature that examines how formal and informal support interact. We show that formal social support does not act alone. It helps to strengthen informal non-economic social support, forming a complementary safety net in background of labor migration and weakening traditional filial norms. Finally, we incorporate a regional heterogeneity to highlight the care insufficient in regions with population outflows. These findings addresses the comparative debates of how demographic shifts, migration, and welfare systems collectively shape social support systems in developing countries.

The structure of the remainder of this paper is as follows: Section 2 presents the theoretical framework for analyzing the impact of formal social support on vulnerability to poverty among rural older adult households, Section 3 describes data sources, variable definitions and empirical strategy, Section 4 reports the empirical results and discussions, and Section 5 concludes the paper and provides policy implications.

## 2 Theoretical framework and research hypotheses

### 2.1 Theoretical framework

Drawing on the concept of ‘vulnerability’ and the World Bank definition of vulnerability to poverty, we define the vulnerability to poverty among rural older adult households as the probability that these households will become poor in the future. Individuals and households face potential risks in the economic, social and natural environments (19). These risks may directly or indirectly reduce their welfare and may push households that are not poor into poverty, worsen the situation of already vulnerable households, or lead to long term poverty. Households often use risk response strategies such as selling assets, taking loans and seeking support from others to reduce potential losses (20). Therefore, vulnerability to poverty is the result of both risk shocks and risk responses. For rural older adult households, their level of capital and the support systems around them shape the risks they face and their ability to respond. These households are especially vulnerable because they have limited human, economic and social capital. As a result, they depend more on social support systems, which becomes a key factor that shapes their vulnerability to poverty.

The risk “shock-response” framework constructed in our study offers a significant complement to Amartya Sen’s Capability Approach (CA) and the Social Determinants of Health framework (SDOH) of WHO. The CA and SDOH frameworks mainly focus on the static factors that lead to vulnerability to poverty among rural older adult households. The risk “shock-response” framework provides an analytical tool for examining the dynamic process through which vulnerability is formed. Specifically, a core idea of the CA is that poverty is not only a lack of income but a “deprivation of capabilities” (21). The risk “shock-response” framework makes this view more dynamic. It shows how existing capabilities are weakened when rural older adult households face risk shocks and how they then use different resources to respond. Furthermore, the SDOH framework states that health is determined by a wide range of social, economic and environmental conditions, rather than merely by access to medical services.^6^ This offers a critical macro background for understanding the vulnerability to poverty among rural older adult households. Our framework explains the micro level processes through which these SDOH factors interact. Within this framework, the “shock” refers to the specific environmental, social, and economic risk factors described in the SDOH. “Response” refers to the key resources, such as economic and social capitals, that households mobilize to response to shocks.

Social support is the perceived or actual receipt of social resources, including objective and emotional support (22). It can be categorized into formal and informal social support according to the source. Formal social support is assistance from formal institutions or organizations, which is part of social security system and is designed to help individuals and households respond to challenges in life. Informal social support is non-professional and non-institutional assistance from family members, friends, neighbors and colleagues. Both formal and informal social support are main factors that reduce the vulnerability to poverty among rural older adult households (6). In our study, formal social support for rural older adult households includes three main parts. These are PSS, MSS and SSS, which refer to pension security, medical security and government assistance, respectively. These programs provide a fundamental livelihood guarantee for rural older adult households. They have wide coverage and are relatively fair in distribution, but they have limited to meet individual needs. Informal social support includes support from adult children and support from relatives and neighbors. These channels provide economic support, such as financial transfers, and non-economic support, such as daily care and emotional support. Within the background of widespread labor migration in China, non-economic forms of informal social support may be even more important for rural older adult households than direct financial transfers.

**Figure 1:**
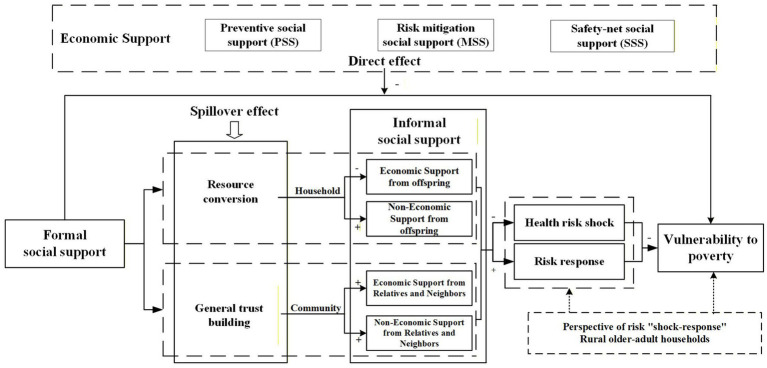
Theoretical analysis framework. Source: Conceptual framework designed by the authors.

Within this framework, formal social support affects the vulnerability to poverty among rural older adult households through two main mechanisms. The first is a direct effect. PSS, MSS and SSS help these households buffer shocks by stabilizing income and reducing out of pocket healthcare expenditure. The second is a spillover effect. Formal social support changes the level and type of informal social support among rural older adult households by influencing budget constraints and social trust. Figure 1 illustrates the mechanism of the impact of formal social support on vulnerability to poverty.

### 2.2 Research hypotheses

Formal social support is a critical factor for rural older adult households to respond to risks and reduce their vulnerability to poverty. The health capital model states that health stock depreciates at a faster rate as people age (23). For rural older adult households, this process manifests as a decline in both physical and cognitive human capital (7), which makes them highly exposed to health shocks. Empirical studies further indicate that long term wear and tear from manual work in rural areas speeds up this decline and raises both the probability and severity of adverse health events (24). Meanwhile, rural older adult households often struggle to meet their basic livelihood through human, natural, or physical capital in the short term. Additionally, they also have limited access to formal financial tools such as loans or commercial insurance because of policy and market constraints (25). In practice, many rural older adult households frequently rely on external support systems to meet their basic livelihood needs. In rural China, formal social support from the government is an important safety net that helps reduce health shocks and stabilizes income. Specifically, medical security support helps rural older adult households pay for necessary health care after a health risk shock, which strengthens their ability to respond to risk and lowers the probability of falling into poverty due to illness. Pension security and government assistance provide relatively stable cash or in-kind transfers that directly mitigate financial pressure and alleviate the poverty among rural older adult households. Based on the analysis, we propose the following research hypothesis:

*Hypothesis* 1: Formal social support has a significant negative impact on the vulnerability to poverty among rural older adult households.

Besides this direct channel, formal social support may also reduce vulnerability to poverty by changing informal social support at both the household and community levels. In this paper, we define the spillover effect of formal social support as behavioral responses of family members, relatives and neighbors to support provided by formal institutions such as the government. Consequently, formal social support changes the structure and level of informal social support available to rural older adult households. Previous studies show that social support can strengthen psychological resilience and mitigate the negative effects of life stress (26, 27). Informal social support has been found to improve physical and mental health of the older adults and reduce health risks (28–30). These findings suggest that if formal social support can effectively shape the structure of informal social support, it may have important implications for health risk shocks and poverty among the rural older adult households.

At the household level, spillovers arise because formal social support relaxes budget constraints and changes intergenerational support duties. When pensions security, medical security and government assistance cover part of basic consumption and health expenditures, adult children are no longer need to provide an equivalent amount of cash transfers to secure the subsistence of rural older adult households. This financial relief allows adult children to reallocate economic support to companionship, daily care and emotional support, which are needed by the rural older adult households. In this sense, formal social support has a spillover effect, whereby economic support from government generate non-economic intergenerational support. Due to the links between informal social support and psychological well-being (31, 32), we believe that the reconfiguration of informal social support can lower the risk of health shocks among rural older adult households and thus reduce their vulnerability to poverty.

At the community level, formal social support improves basic health and living security for the older adults and share the costs of unexpected shocks. This reduces the need for neighbors to provide emergency loans and shifts relations between rural older adult households from one-time economic assistance toward more routine and mutual exchanges. Beyond providing direct economic security for rural older adult households, formal social support significantly reinforces informal social support networks within the community (33).

Previous studies show that in countries with universal welfare systems, citizens report higher levels of civic engagement, voluntary participation and social trust (34). Thus, we argue that formal social support serves as a clear signal of the government commitment to older adult welfare. This commitment builds trust in formal institutions and supports a cooperative social environment, which in turn enhances community cohesion and interpersonal trust. Empirical evidence further confirms that communities with broader pension coverage or health insurance coverage tend to maintain more robust informal support networks (11). Consequently, this complementary relationship creates an important spillover effect through which formal social support further reduces vulnerability to poverty among rural older adult households. Based on this analysis, we propose the following research hypothesis:

*Hypothesis* 2: Formal social support mitigates vulnerability to poverty among rural older adult households not only directly but also through a positive spillover mechanism that crowds in non-economic support from informal social networks.

*Hypothesis* 2a: At the household level, formal social support operates through a resource-release mechanism, enabling adult children to provide emotional support, thereby reducing health risk shocks among rural older adult households.

*Hypothesis* 2b: At the community level, formal social support fosters social capital and reciprocity, thereby improving risk response capacity among rural older adult households.

## 3 Study design

### 3.1 Data source and participants

This study uses data from three waves of the CFPS in 2018, 2020 and 2022. The CFPS is conducted by Institute of Social Science Survey at Peking University. It is a national survey at the individual, family and community levels and provides rich information on the economic and non-economic welfare of Chinese residents. The CFPS covers more than 95 percent of the population in 25 provinces, municipalities and autonomous regions in China. It includes key information for the study of vulnerability to poverty among rural older adult households, such as demographic characteristics, health condition, medical and pension insurance, and household income and assets. The CFPS is is widely regarded as reliable and it offers a sound basis to examine the link between social support and vulnerability to poverty among rural older adult households in China. Furthermore, 2020 was the last year of China’s targeted poverty alleviation campaign. Using CFPS data from 2018 to 2022 allows us to provide timely evidence on the vulnerability to poverty among rural older adult households and to assess the effects of poverty alleviation policies and policies that aim to prevent a return to poverty. The sample selection in this study follows three steps: (a) we identified households in which the householder was aged 60 years or older. These households were defined as older adult households; (b) we restricted the sample to households with rural registration status; and (c) we removed observations with outliers or missing values in key variables. The final sample includes 3,258 rural older adult households. We follow a rigorous procedure to process data. First, we constructed an unbalanced panel to keep as many observations as possible and to reduce attrition bias. Second, we adopted a multi-stage imputation strategy to address missing values in key variables such as household consumption, net assets and pension or retirement. Specifically, we performed logical imputation based on related household characteristics, followed by a combination of multiple imputation and mean imputation to complete the dataset. Finally, since income and consumption variables showed a strong right tail, we winsorized these variables at the top and bottom 1% to mitigate the influence of extreme values. Subsequently, these variables were log-transformed to approximate a normal distribution. As CFPS has been approved by the Peking University Biomedical Ethics Committee (approval number: IRB00001052-14010) and is open to the whole community, no additional ethical approval is needed.

### 3.2 Variables and statistical description

#### 3.2.1 Dependent variable: vulnerability to poverty

The dependent variable is vulnerability to poverty. The measurement of vulnerability to poverty has been extensively studied from different perspectives, mainly including risk and the welfare perspective. Research from the risk perspective emphasizes the impact of risk shocks on individuals or households’ welfare and derives the concepts of vulnerability to risk exposure (VER) and vulnerability to expected utility (VEU). VER measures welfare sensitivity to risk shocks (35), while VEU focuses on the possibility that welfare expectations will fall below the poverty line under conditions of uncertainty (36). In contrast, the welfare perspective introduces the concept of vulnerability as expected poverty (VEP), which defines vulnerability as the possibility that future welfare levels of households will fall below the poverty line (37). The VEP method has been widely used in many studies due to its operationalizability and ability to provide prospective analysis when longitudinal data are insufficient (37–40). We agree with the applicability of the VEP for measuring the vulnerability to poverty among rural older adult households.

In our study, the core concept of VEP is the probability of households falling below the poverty line in a certain period in the future, based on their current welfare levels (measured by income or consumption) and the distribution characteristics of welfare level. Furthermore, we incorporate variables capturing risk shocks and risk response into the measurement model of VEP to extend and refine its framework. The basic predictive Equation 1 is expressed as follows:


(1) $$\mathrm{VE}P_{h,t}=\mathrm{Pr}(Y_{h,t+1}⩽L)$$

where $\mathrm{VE}P_{h,t}$ represents the vulnerability to poverty among household h in period t, Pr(·) denotes the probability that the household’s future income or consumption level $Y_{h,t+1}$ will fall below the poverty line *L*.

Thus, $\mathrm{VE}P_{h,t}$ measures the probability that a household’s per capita consumption expenditures will drop below the poverty line, reflecting its probability of falling into poverty in the future. We assume that future welfare $Y_{h,t+1}$ follows a lognormal distribution, the measurement equation for $\mathrm{VE}P_{h,t}$ can be further specified as follows:


(2) $$\mathrm{VE}P_{h,t}=\int_{-\infty }^{\mathrm{lnL}}\;f_{t}(\ln Y_{h,t+1})d(\ln Y_{h,t+1})=\emptyset (\frac{\mathrm{lnL}-\mu_{h}}{\sigma_{h}})$$

where $\mu_{h}$ and $\sigma_{h}$ denote the expected value and standard deviation of future welfare, respectively. As indicated by Equation 2, accurate estimation of future welfare ( $Y_{h,t+1}$ ) is crucial for obtaining precise measurements of $\mathrm{VE}P_{h,t}$ . However, most studies incorporate risk factors in the error term of the model, and this method potentially overlooks the direct impact of risk shocks on rural older adult households, especially the situation of poverty due to the lack capacity of risk response. Therefore, it may lead to biased welfare estimates if neglects the influence of health risk shocks and risk response capacity. To address this limitation, we referred to the VEP model by incorporating a risk “shock-response” perspective to households. The improved welfare model is expressed as follows:


(3) $$\ln Y_{h,t}=a_{0}+X_{h,t}^{\prime }a+a_{1}R_{h,t}+a_{2}D_{h,t}+e_{h,t}$$

where $X_{h,t}^{\prime }$ includes independent variables affecting welfare, such as (age, age square, marriage condition, health condition, per capita income, household size, co-residence, land, agricultural production), $R_{h,t}$ represents household risk shocks in period t , $D_{h,t}$ denotes the household’s risk response capacity in period t , and $e_{h,t}$ is the error term in period t . To measure vulnerability to poverty from a risk “shock-response” perspective, we adopt a three-stage Feasible Generalized Least Squares (3FGLS) approach, specified as follows:

First, the welfare equation (Equation 3) is estimated to derive residual term $e_{h,t}$ and obtain predicted per capita welfare measures for older adult households.

Second as indicated by Equations 4, 5, we compute both the expected value $\hat{Y}$ and variance $\sigma_{e,h}^{2}$ of per capita consumption for older adult household, specified as follows:


(4) $$\hat{Y}={\hat{a}}_{0}+X_{h}^{\prime }\hat{a}+R_{h}{\hat{a}}_{1}+D_{h}{\hat{a}}_{2}$$

(5) $$\sigma_{e,h}^{2}={\hat{\beta }}_{0}+X_{h}^{\prime }\hat{\beta }+R_{h}{\hat{\beta }}_{1}+D_{h}{\hat{\beta }}_{2}$$

Third, we assume that the consumption of older adult household follows a lognormal distribution, use Equation 2 to calculate the VEP. The resulting VEP index ranges from 0 to 1, with higher values indicating greater vulnerability to poverty among rural older adult households.

Notably, we use consumption rather than income to measure welfare because income may be endogenous in the model. Meanwhile, consumption is typically less volatile and better reflects household welfare (41, 42). For the baseline analysis, we adopt the World Bank consumption standard, which defines the poverty line as US$1.90 per person per day. In practice, we adjust the poverty line according to 2018, 2020 and 2022 PPP and province level rural Consumer Price Index (CPI) in the actual calculation of VEP, with $\mathrm{VE}P_{1.9}$ . For robustness checks, we also use a higher poverty line at US$3.10 per day per person to calculate $\mathrm{VE}P_{3.1}$ . Following the approaches of previous studies, we set vulnerability thresholds at 0.5 and 0.29 (43, 44). Rural older adult households with a probability of falling into poverty exceeding 50% or 29% are defined as vulnerable older adult households, and we construct a binary variable based on this definition for robustness checks.

#### 3.2.2 Independent variable: formal social support

Formal social support is categorized into PSS, MSS and SSS. PSS is measured by pensions or retirement benefits received among rural older adult households over the past year, calculated from the questionnaire item, “*Amount of pension/retirement benefits received by your household in the last 12 months*.” MSS is measured by the enrollment rate in the New Rural Cooperative Medical Scheme among adults in the household. SSS is measured by government assistance received among rural older adult households over the past year, based on the questionnaire item, “*Including cash and in-kind subsidies, what was the total value of government grants received by your household in the last 12 months*.” This design is based on two considerations. First, in rural China, the support of the rural older adult households from formal institutions is primarily delivered through medical security, pension security and government assistance programs. Previous studies measure formal social support using pensions and medical insurance and explicitly include other government assistance as part of the same institutional pillar when they study health and well-being, which aligns with our approach (45). Second, from the perspective of preventing vulnerability to poverty, each of the three components plays an essential and non-substitutable role. For rural older adult households, pension security provides a stable and predictable income floor and has an important preventive function. Medical security is essential for mitigating the results of health risk. Government assistance provides basic subsistence support for daily living and acts as a safety net. The absence of any one component can greatly increase the risk of poverty. We therefore treat all three pillars as equally important for preventing vulnerability to poverty among rural older adult households.

#### 3.2.3 Mechanism variable: informal social support

To examine the spillover effects of formal social support, we divide informal social support into four components, including economic and non-economic support from both household and community. At the household level, economic support from adult children (*CESS*) is measured by the transfer amount from adult children, calculated from the questionnaire item, “*In the past 12 months, how much financial or in-kind support did your household receive from adult children (not residing in the same house)?*.” Non-economic support from adult children (*CSS*) is measured using principal component analysis to construct a comprehensive index of daily care (Care), emotional comfort (Emotional), daily interactions (Interaction) and the quality of the relationship with children (Relationship). These dimensions are based on responses to the following questionnaire items *“Over the past 6 months, how often have you been able to see your adult children?”* “*Have your adult children taken care of your household chores or daily needs in the past six months?*” “*Over the past 6 months, how often have you been in contact with your adult children via phone calls, text messages, letters, or emails?*” and “*How has your relationship with your adult children been over the past 6 months?*”

Prior to conducting factor analysis, we examined the correlation structure of the variables to assess whether the data were suitable for dimension reduction. The preliminary correlation analysis shows that many variables exhibit strong correlations. We performed the Kaiser Meyer Olkin (KMO) test and Bartlett’s test of sphericity for further statistical validation. As shown in Table 1, the KMO measure of sampling adequacy is 0.677. This value exceeds the commonly accepted threshold of 0.6 and indicates acceptable sampling adequacy. Furthermore, Bartlett’s test yields a *p* value of 0.000, which is statistically significant at the 1% level. This result allows us to reject the null hypothesis that the variables are orthogonal. Collectively, these tests confirm that the data on non-economic support from adult children variable are suitable for factor analysis.

**Table 1:** Results of KMO test and Bartlett spherical test.

Variable	Factor loading	Communality
Care	0.792	0.627
Emotional	0.617	0.381
Interaction	0.769	0.592
Relationship	0.819	0.672
Eigenvalue	2.271	
Proportion	56.8%	
Cumulative	56.8%	
Chi-square	3437.912	
Degrees of freedom	6	
*p*-value	0.000	
KMO	0.677	

Similarly, at the community level, economic support from neighbors (*NESS*) is quantified based on reported financial or in-kind support from neighbors. Non-economic support from neighbors (*NSS*) is measured by the degree of trust in neighbors, based on the questionnaire item *“How much trust do you have in your neighbor?,”* where 0 points indicate *very distrust*, 10 points indicate *very trust*.

#### 3.2.4 Control variables

Considering the factors influencing vulnerability to poverty among rural older adult households, we incorporate control variables based on previous studies, including demographic and socioeconomic characteristics of the household head and household. Specifically, the study considered age (*Age*), age square (*Age2*), marital status (*Mar*), number of adult children (*Child*), health condition (*Health*), dependency ratio (*Ratio*), co-residence (*Res*) and land (*Land*). Descriptive statistics for these variables are shown in Table 2.

**Table 2:** Descriptive statistics of variables (Obs. = 3,258).

Variable	Description	Mean	SD	Min	Max
*VEP*_1.9_	Vulnerability to expected poverty	0.123	0.127	0	0.963
PSS	Pension income (Unit: yuan in logarithm form)	5.866	3.69	0	10.94
MSS	Medical security coverage (Unit: %)	0.741	0.333	0	1
SSS	Government subsidy (Unit: yuan in logarithm form)	3.53	3.609	0	9.575
CESS	Economic support from adult children (Unit: yuan in logarithm form)	3.244	3.925	0	10.309
CSS	Non-economic support from adult children (Index)	0	1.507	−3.468	2.429
NESS	Economic support from neighbors (Unit: yuan in logarithm form)	0.664	2.027	0	8.412
NSS	Trust in neighbors (Score: 0–10)	6.622	2.758	0	10
Age	Age of the householder (Unit: years)	68.448	5.982	60	93
Age2	Age squared	47.209	8.466	36	86.49
Mar	Marital status (Married = 0, otherwise = 1)	0.760	0.427	0	1
Child	Number of adult children (Unit: count)	1.010	0.945	0	9
Health	Self-rated health (Score: 1–5)	2.607	1.284	1	5
Ratio	Old-age dependency ratio (Unit: %)	2.537	2.701	0	6
Res	Co-residence with adult children (1 = yes)	0.318	0.466	0	1
Land	Having agricultural land (1 = yes)	0.848	0.359	0	1

The table presents summary statistics for all variables, including the mean, standard deviation, minimum, and maximum values. *VEP*_1.9_ represents vulnerability to poverty calculated using the poverty line US$1.90. Data source: 2018–2022 waves of CFPS.

Table 2 presents the descriptive statistics of the variables. The average probability of falling into poverty among rural older adult households is 0.123 when using a poverty line US$1.90 per day per person. This suggests that most rural older adult households face a risk of future poverty in China. The remaining part of Table 2 presents detailed statistics of the control variables.

Regarding individual characteristics of older adult householders, the average age exceeds 68 years. Nearly 76% of the householders in the sample are married, suggesting relatively stable household structures. The self-reported health condition of householders is generally poor, indicating high vulnerability to health-related risk shocks. On average, rural older adult households have at least one adult child, reflecting potential access to informal social support from adult children. Moreover, 31.8% of older adult households do not live with their children and 84.8% engage in agricultural production.

**Figure 2:**
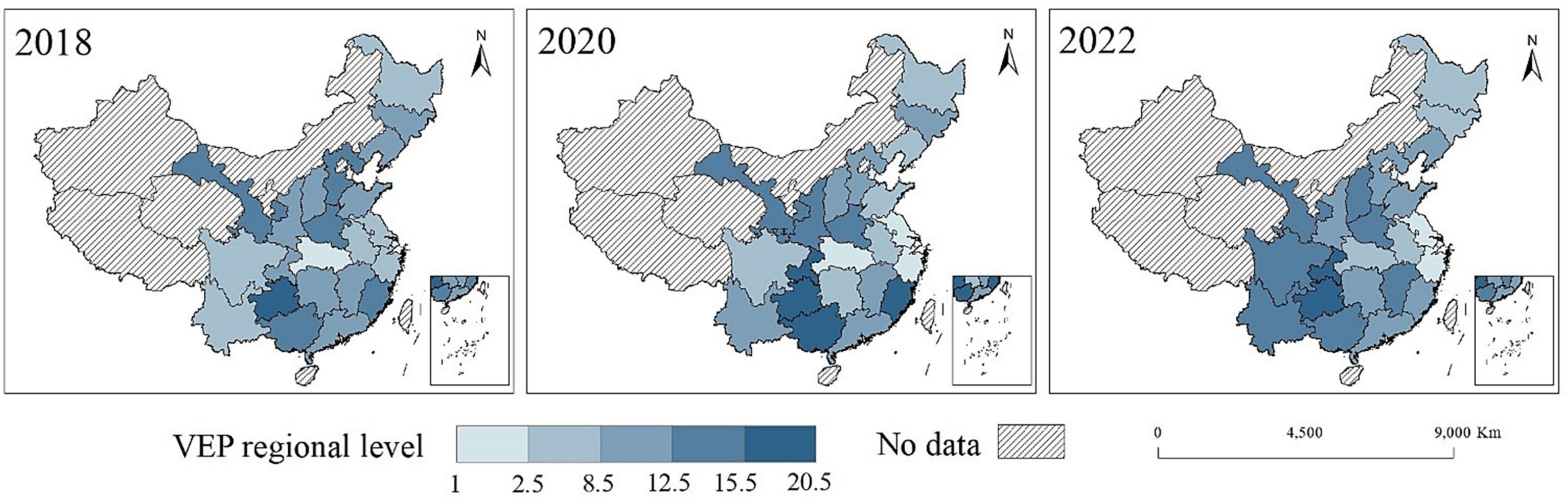
Spatial distribution of vulnerability to poverty among rural older adult households in China at the US$1.90/day poverty line, 2018–2022. Data source: 2018–2022 waves of CFPS. This map is based on the standard map of the Ministry of Civil Affairs of the People’s Republic of China [Review No. GS (2020)4619] (http://bzdt.ch.mnr.gov.cn/index.html).

Figure 2 shows the spatial distribution of vulnerability to poverty among rural older adult households in 2018, 2020 and 2022, using the international poverty line of US$1.90 per person per day as the poverty threshold. Overall, the maps show a clear east and west gradient. Provinces in the eastern coastal region generally exhibit lower VEP levels, while a continuous belt of high VEP appears in the less developed central and western provinces. From 2018 to 2020, this high-risk belt expands and deepens, and several central and southern provinces shifting from medium to high vulnerability, which suggests that the risk of falling into poverty intensified in the study period among rural older adult households. By 2022, VEP falls slightly in some provinces, but high vulnerability remains concentrated in much of the central, southwestern and parts of the southern region, indicating that the spatial pattern of poverty risk is remarkably persistent. In addition, the northeastern provinces show a gradual rise in VEP over time, approaching the risk level in the central and western regions. This increasing vulnerability in the northeast may be related to sustain out migration of younger labor and the associated weakening of family and community support for the rural older adults.

### 3.3 Model specification

To estimate the impact of formal social support on vulnerability to poverty among rural older adult households, using Equation 6, we first constructed the following fixed effect model:


(6) $$\mathrm{VE}P_{\mathrm{hjt}}=\lambda_{0}+\lambda_{1}\mathrm{PS}S_{\mathrm{hjt}}+\lambda_{2}\mathrm{MS}S_{\mathrm{hjt}}+\lambda_{3}\mathrm{SS}S_{\mathrm{hjt}}+X_{\mathrm{hjt}}^{\prime }\lambda +\varepsilon_{\mathrm{ijt}}$$

Where, $\mathrm{VE}P_{\mathrm{hjt}}$ represents the vulnerability to poverty among rural older adult household h at t year in j region and serves as the dependent variable; $\mathrm{PS}S_{\mathrm{hjt}}$ , $\mathrm{MS}S_{\mathrm{hjt}}$ , and $\mathrm{SS}S_{\mathrm{hjt}}$ denote the independent variable; $X\prime_{\mathrm{hjt}}$ represents a vector of control variables. $\lambda_{0}$ is the constant term; $\lambda_{1}$ , $\lambda_{2}$ and $\lambda_{3}$ represent the parameter of interest, and $\varepsilon_{\mathrm{ijt}}$ is the random error term.

We further investigate the spillover effects of formal social support and its role in alleviating vulnerability to poverty among rural older adult households. Previous empirical studies on spillover effects use several approaches, including Spatial Durbin Models (46), difference in differences models (DID), fixed effects models (FE), random effects models (RE), and observational regression methods (47). Spatial Durbin Models are employed to analyze spatial spillovers. DID, FE, and RE models are generally used to test for spillover effects with panel data. Observational regression methods are used to examine institutional spillovers in cross-sectional data (21). Given that our data are panel data, we follow the fixed effects approach for the empirical analysis. As indicated by Equation 7, the model is specified as follows:


(7) $$\text{Informa}l_{\mathrm{hjt}}=\delta_{0}+\delta_{1}\text{Formal}\_\text{Suppor}t_{\mathrm{hjt}}+X_{\mathrm{hjt}}^{\prime }\delta +\varepsilon_{\mathrm{ijt}}$$

Where, $\text{Informa}l_{\mathrm{hjt}}$ is the mechanism variable, representing informal social support and including NSS, NESS, CSS and CESS. The coefficient $\delta_{1}$ captures the spillover effect of formal social support. A significant $\delta_{1}$ would indicate that formal social support positively influences informal social support.

## 4 Empirical results and discussion

### 4.1 Baseline results

#### 4.1.1 Multicollinearity test

Variance Inflation Factor (VIF) is one of the methods used to detect multicollinearity among variables in the model. Generally, a VIF value ≥5 indicates significant multicollinearity, while a VIF value ≥10 suggests a high degree of multicollinearity. As shown in Table 3, all the independent variables in this study have VIF values <5, and the mean VIF is 1.12, indicating that the model does not suffer from multicollinearity issues and is well-constructed.

**Table 3:** Results of the multicollinearity test (Obs = 3,258).

Variable	*VEP*1.9 (OLS)		Collinearity statistics	
	B (Significance)	Std. Error	Tolerance	VIF
PSS	−0.495^***^	(0.065)	0.970	1.03
MSS	−2.952^***^	(0.691)	0.953	1.05
SSS	−0.194^***^	(0.058)	0.941	1.06
Age	0.349^***^	(0.042)	0.807	1.24
Marriage	−3.057^***^	(0.553)	0.913	1.09
Health	−4.455^***^	(0.171)	0.986	1.01
Ratio	−0.542^***^	(0.084)	0.847	1.18
Child	1.385^***^	(0.286)	0.738	1.35
Res	−2.604^***^	(0.533)	0.736	1.36
Land	3.521^***^	(0.692)	0.933	1.07
Cons.	−4.461	(3.401)	/	/

*VEP*_1.9_ is the vulnerability to poverty calculated based on the poverty line standard of US$1.90; ***, **, and * indicate significance levels at 1, 5, and 10%, respectively; Standard errors in parentheses. Data source: 2018–2022 waves of CFPS.

#### 4.1.2 Fixed effect model results

As shown in Table 4, we estimate the impact of formal social support on the vulnerability to poverty among rural older adult households, using international poverty line of US$1.90 per person per day. Columns (1) reports the baseline results without control variables, while Columns (2) adds the control variables. The estimated coefficients for PSS, MSS and SSS are −0.314, −3.115 and −0.258, respectively. All of them statistically significant at the 1% level. This indicates that a robust negative association between formal social support and vulnerability to poverty among rural older adult households. However, *p* values alone are not sufficient to assess the substantive importance of formal social support. We further assess the effect size by calculating the standardized Beta coefficients. The standardized Beta coefficients for PSS and SSS in Column (2) are approximately −0.314 and −0.258. These results suggest that the average vulnerability to poverty among older adult households falls by approximately 0.314 and 0.258 percentage points when PSS and SSS doubles. Thus, these findings suggest that PSS and SSS are not only statistically significant but also constitutes a substantively important factor in mitigating the vulnerability to poverty among rural older adult households, which provides stronger and more credible support for hypothesis H1.

**Table 4:** Baseline results.

Variable	(1) *VEP*1.9	(2) *VEP*1.9
PSS	−0.300^***^	−0.314^***^
	(0.084)	(0.083)
MSS	−3.194^***^	−3.115^***^
	(0.998)	(1.012)
SSS	−0.243^**^	−0.258^***^
	(0.098)	(0.098)
Age		−1.535
		(1.776)
Age2		0.805
		(1.199)
Mar		0.630
		(2.258)
Health		0.039
		(0.271)
Child		0.133
		(0.158)
Ratio		−1.633
		(1.313)
Res		−1.677
		(1.032)
Land		1.535
		(1.023)
Year FE	No	Yes
Household FE	No	Yes
Region FE	Yes	Yes
Cons.	35.208^***^	102.084
	(1.299)	(68.499)
Obs.	3,258	3,258
R-squared	0.069	0.074

*VEP*_1.9_ is the vulnerability to poverty calculated based on the poverty line standard of US$1.90; ***, **, and * indicate significance levels at 1, 5, and 10%, respectively; Standard errors in parentheses. Data source: 2018–2022 waves of CFPS.

### 4.2 Robustness test

#### 4.2.1 Replacing the dependent variable

In addition to utilizing the US$1.90 per person per day standard in the baseline regression, we introduce additional absolute poverty line standards to recalculate the vulnerability to poverty among rural older adult households. Subsequently, the robustness of the regression results is tested. The US$1.90 per day standard is replaced with the World Bank’s absolute poverty line standard of US$3.10 per person per day (2011 PPP) for lower middle-income countries (48). Therefore, Equation 2 is employed to re-evaluate the vulnerability to poverty among rural older adult households, yielding estimation results presented in Column (1) of Table 5. The PSS, MSS and SSS significantly mitigate the vulnerability to poverty among rural older adult households, irrespective of the chosen absolute poverty line for measuring such vulnerability.

**Table 5:** Robustness test results.

Variable	(1)	(2)	(3)	(4)	(5)
	*VEP*3.1	*VEP*1.9	$\mathrm{VE}P_{1.9}^{1}$	$\mathrm{VE}P_{1.9}^{2}$	*VEP*1.9
PSS	−0.381^***^	−1.899^***^	−0.006^***^	−0.003^***^	−0.344^***^
	(0.098)	(0.651)	(0.001)	(0.001)	(0.099)
MSS	−3.723^***^	−2.545^**^	−0.051^***^	−0.013^**^	−2.812^*^
	(1.202)	(1.132)	(0.015)	(0.006)	(1.445)
SSS	−0.306^***^	−1.491^**^	−0.005^***^	−0.002^***^	−0.214^**^
	(0.114)	(0.692)	(0.001)	(0.001)	(0.104)
Cons.	178.971^*^	121.791^*^	/	/	49.721
	(95.037)	(68.811)	/	/	(77.877)
Controls	YES	YES	YES	YES	YES
Year FE	YES	YES	YES	YES	YES
Household FE	YES	YES	YES	YES	YES
Region FE	YES	YES	YES	YES	NO
Obs.	3,258	3,258	3,258	3,258	3,080
R-squared	0.110	0.064	/	/	0.071

$\mathrm{VE}P_{1.9}^{1}$ is the vulnerability to poverty calculated based on the poverty line standard of US$1.90 and 29% vulnerability line; ${\mathrm{VEP}}_{1.9}^{2}$ is the vulnerability to poverty calculated based on the poverty line standard of US$1.90 and 50% vulnerability line. The estimated models in (1) ~ (2) and (3) ~ (4) are conditional fixed effects model and fixed effects logit model, respectively. The estimated models in (5) is weighted panel fixed-effects model. The key independent variables in the (2) is measured as 0–1 variables; ***, **, and * indicate significance levels at 1, 5, and 10%, respectively; Standard errors in parentheses. Data source: 2018–2022 waves of CFPS.

#### 4.2.2 Replacing the measure of the independent variable

We re-quantified PSS, MSS and SSS based on whether rural older adult households receive pension/retirement benefits, medical security and government assistance, respectively assigning a value of 1 if they do and 0 otherwise. Table 5 displays the results in Column (2), showing that rural older adult households with PSS, MSS and SSS are less likely to experience poverty. These results are consistent with the baseline regression, further validating the robustness of our earlier findings.

#### 4.2.3 Replacing the empirical model

Following the previous study (49), we construct a dummy variable for vulnerability to poverty, assigning a value of 1 to households with vulnerability levels equal to or above the vulnerability threshold (0.29 or 0.5), and estimate its effect using a Conditional Fixed Effects Logit Model. The results are shown in Columns (3) and (4) of Table 5, reporting the marginal effects of PSS, MSS and SSS on vulnerability to poverty among rural older adult households under the US$1.90 poverty line at vulnerability thresholds of 0.29 ( $\mathrm{VE}P_{1.9}^{1}$ ) and 0.5 ( $\mathrm{VE}P_{1.9}^{2}$ ), respectively. In all cases, the estimated coefficients of PSS, MSS and SSS are significant negative, consistent with the baseline regression results. These findings confirm that formal social support could be associated with reduced likelihood of poverty among rural older adult households.

#### 4.2.4 Using samples weights

To verify that our results are not driven by sampling biases or sample loss, we re-estimated the models using longitudinal weights. Given the CFPS’s multi-stage, stratified, and PPS sampling design, along with the issue of panel attrition, weighting is crucial for maintaining the national representativeness of the rural older adult household subsample. In this specification, the individual longitudinal weight of the household head serves as the weighting variable for the household level analysis. The results present in Column (5) of Table 5, showing that the coefficients of PSS, MSS and SSS of the impact on vulnerability to poverty among rural older adult households are significant negative, which consistent with the baseline regression results.

#### 4.2.5 Endogenetic treatment

While the fixed effects specification mitigates bias driven from time invariant confounders, it does not account for simultaneity or reverse causality. A primary concern is that older adult households exhibiting higher vulnerability are inherently more likely to receive formal social support, potentially biasing the fixed effects estimates. To address this endogeneity and support a causal interpretation, we use an instrumental variable approach.

In this study, PSS, MSS and SSS are proxied by pension or retirement benefits, health insurance participation rate and government assistance, respectively. Eligibility for pensions or retirement benefits is strictly constrained by the biological characteristic of age (60 years old) rather than household economic status. Additionally, pension or retirement benefits levels are largely determined by historical contribution records or uniform local government subsidies. Therefore, PSS is a predetermined variable that is not directly affected by current household economic shocks. In rural China, basic medical insurance is almost universal and has a near mandatory design, so households have limited choice over whether to enroll. This feature largely mitigates self-selection bias. Furthermore, our inclusion of household fixed effects effectively absorbs time invariant unobserved heterogeneity, such as risk preferences. Consequently, MSS can be treated as relatively exogenous. In contrast, government assistance is typically means tested and depends on economic status, leading to a pronounced reverse causality between the receipt of government assistance and the vulnerability to poverty among rural older adult households.

Following the previous literature (50, 51), we use a community level “leave one out” mean of SSS as an IV to address potential endogeneity. On the one hand, social security policies are usually implemented at the local level in China. The rural older adult households in the same community face similar policy settings and administrative conditions, which creates a strong link between individual benefit receipt and the community average. On the other hand, SSS at the community level coverage is an aggregate outcome driven by macro policy factors and is unlikely to be influenced by any single household. Furthermore, the SSS status of neighbors should not directly affect vulnerability to poverty among a given household through channels other than the spillover of formal social support.

As anticipated, the results of the first-stage regression, presented in columns (1) of Table 6, demonstrate a significantly positive coefficient on the IV. In columns (2), the results of the unidentifiable test and Wald χ^2^ is 21.193, with a p value = 0.000, which is <0.01, strongly rejecting the null hypothesis that the instrumental variables are unidentifiable. Furthermore, the Cragg-Donald Wald F-statistics in columns (1) is reported as 21.344, respectively, both exceeding a threshold of 10, further excluding the issue of weak instrumental variables. Column (2) reports the results of the second stage regression, supporting the baseline regression findings that SSS significantly reduces vulnerability to poverty among rural older adult households.

**Table 6:** Endogeneity test results.

Variable	First stage	Second stage
	(1)	(2)
SSS		−0.013^*^
		(0.007)
IV_SSS	0.179^***^	
	(0.039)	
PSS		−0.003^***^
		(0.001)
MSS		−0.033^***^
		(0.011)
Cons.	22.220	1.068
	(26.889)	(1.213)
Controls	YES	YES
Year FE	YES	YES
Household FE	YES	YES
Cragg-Donald Wald F	21.344	/
Unidentifiable test	/	21.193
Wald χ^2^	/	4796.52
*Obs.*	2,767	2,767
adj. *R*^2^	0.061	/

***, **, and * indicate significance levels at 1, 5, and 10%, respectively; Standard errors in parentheses. Data source: 2018–2022 waves of CFPS.

### 4.3 Heterogeneity analysis

**Figure 3:**
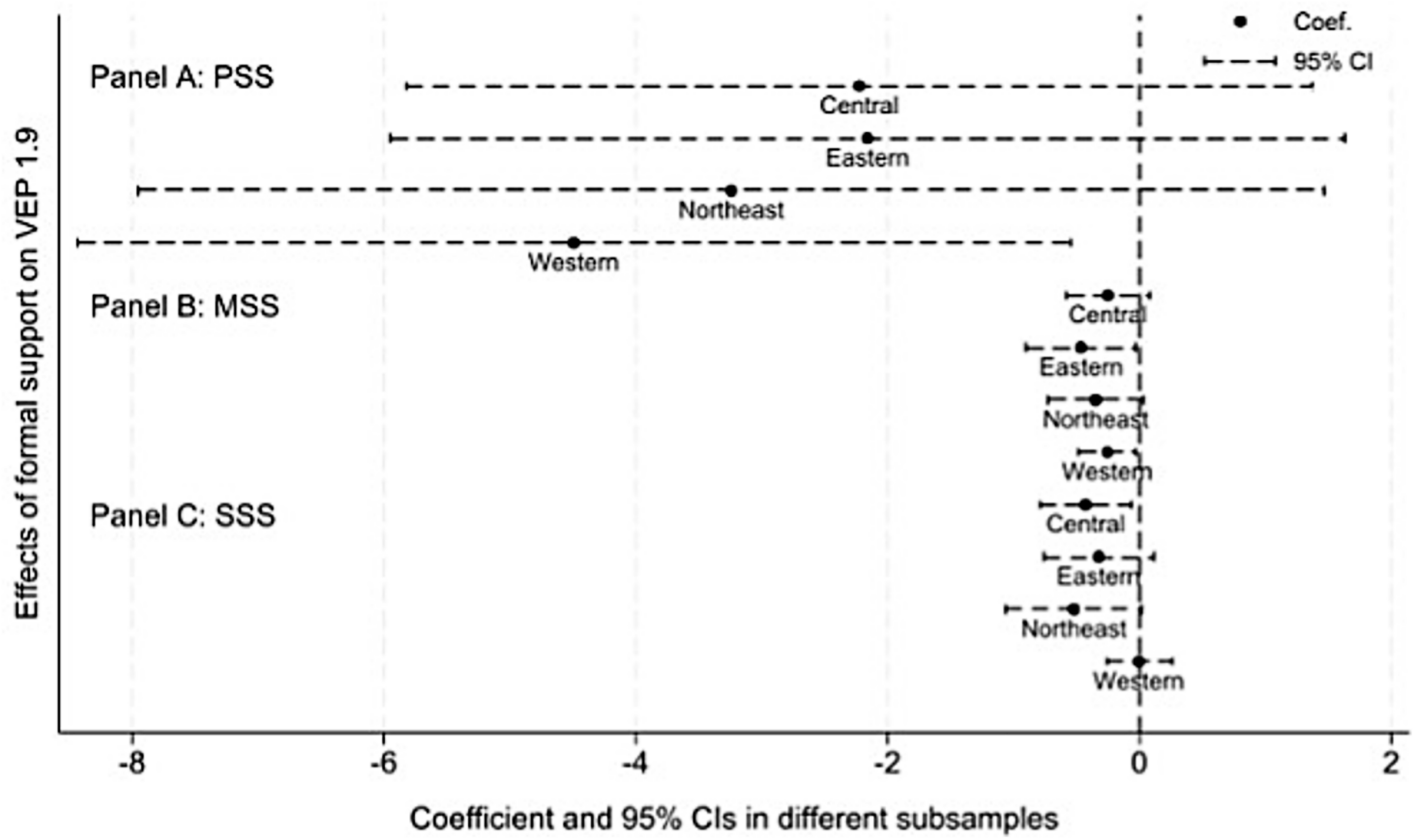
Heterogeneous effects of formal social support on VEP among rural older adult households, by region. *VEP*_1.9_ is measured under the US$1.90 poverty line standard. Data source: 2018-2022 waves of CFPS.

**Figure 4:**
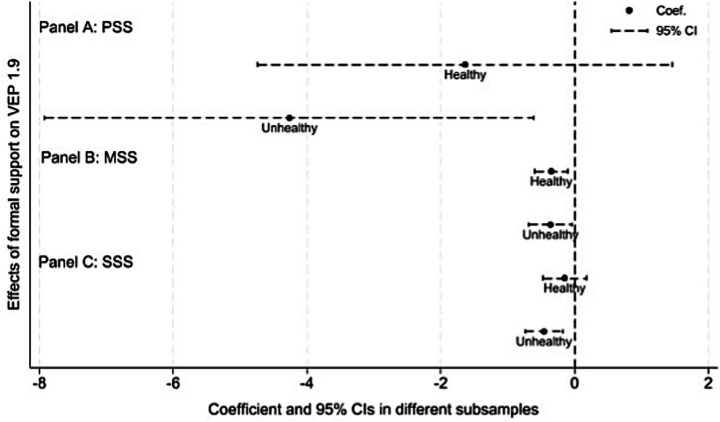
Heterogeneous effects of formal social support on VEP among rural older adult households, by health status. *VEP*_1.9_ is measured under the US$1.90 poverty line standard. Data source: 2018-2022 waves of CFPS.

**Figure 5:**
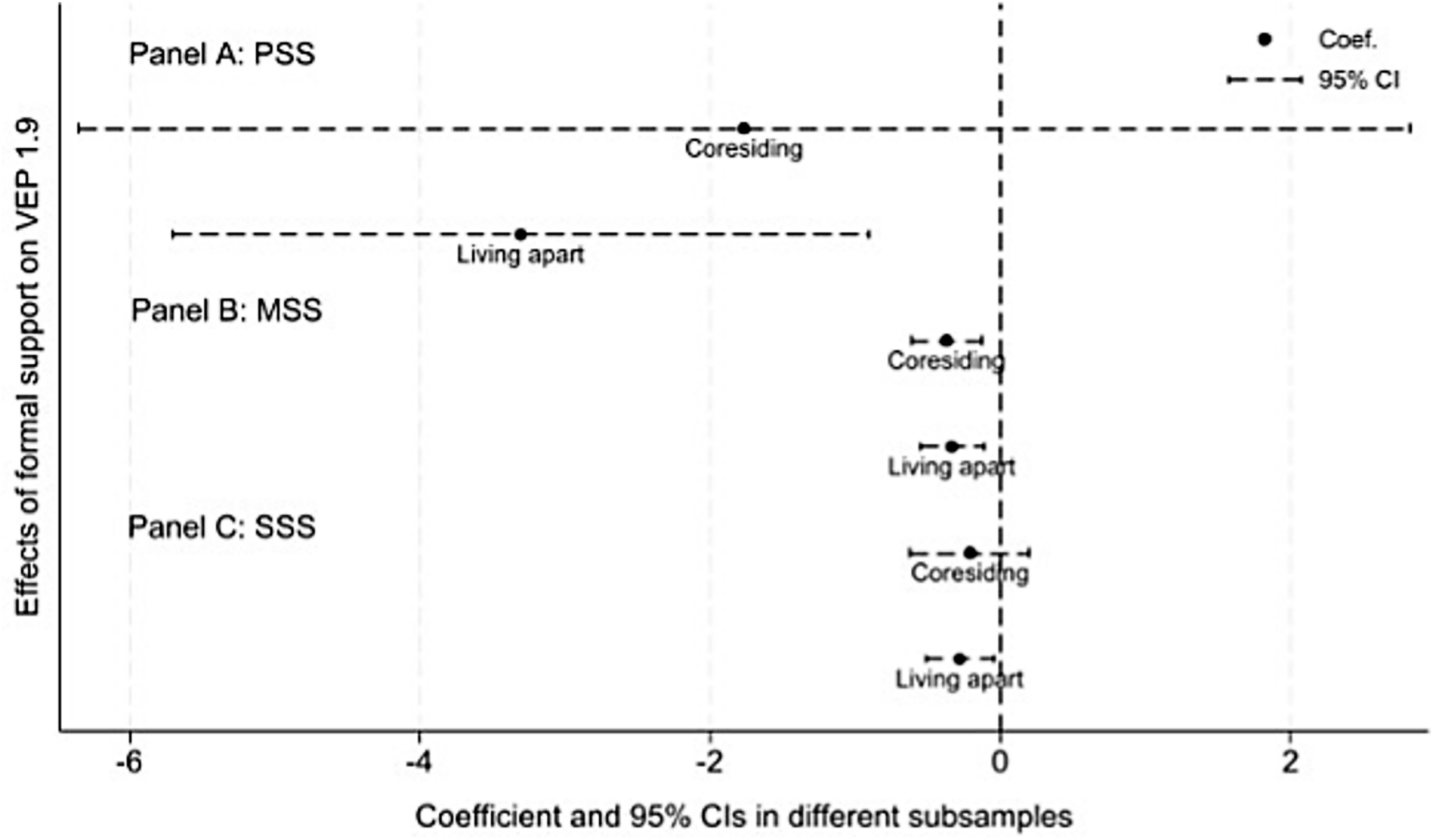
Heterogeneous effects of formal social support on VEP of rural older adult households, by living arrangement. *VEP*_1.9_ is measured under the US$1.90 poverty line standard. Data source: 2018-2022 waves of CFPS.

We proceed to examine the heterogeneity in the impact of formal social support across different regions, health conditions, and living arrangements. We refer to the method of previous research, the visualizations in Panels A, B, and C of Figures 3–5 provide a clear and intuitive comparison of these differences (52).

#### 4.3.1 Differences in region

To further examine the regional heterogeneity in the effects of formal social support on reducing poverty, we divide the sample into four subsamples, including eastern, central, western and northeastern China. Then we re-estimate the baseline specification for each subsample. The results are reported in Figure 3, which plots the regression coefficients of the three types of formal social support (PSS, MSS and SSS) and their 95% confidence intervals for different regions.

The estimates indicate pronounced regional heterogeneity in the effect of PSS on reducing vulnerability to poverty among rural older adult households, displaying a clear “high in the West and low in the East” pattern. In relatively less developed western and central regions, where rural older adult households have weaker risk response capacity, PSS constitutes an important supplement to household income and thus exhibits much stronger marginal effects than in the more developed eastern region. By contrast, MSS significantly reduces vulnerability to poverty among rural older adult households in the eastern region, while its effects are insignificant in the other three regions. This pattern likely reflects the still limited coverage and effectiveness of medical security in the central, western and northeastern regions. This prevents MSS from fully playing its poverty-preventing and risk-mitigating role in less developed areas. In addition, SSS significantly reduces vulnerability to poverty among rural older adult households in the central region, suggesting that government assistance has played an important role in poverty reduction in central China.

#### 4.3.2 Differences in health condition

Health risk shocks are a key factor driving rural older adult households into poverty, we split the sample into “healthy” and “unhealthy” groups according to the health condition of the household head and re-estimate the model separately for each group. Figure 4 reports the estimated coefficients of PSS, MSS and SSS on vulnerability to poverty among rural older adult households, together with their 95% confidence intervals, for the two health conditions groups.

Panels A and C of Figure 4 reveal heterogeneity in the effects of PSS and SSS on vulnerability to poverty across health groups. For the “unhealthy” group, the coefficients on PSS and SSS are significantly negative, indicating that these two forms of formal social support play a crucial safety-net role for rural older adult households in poor health, significantly reducing their risk of falling into poverty. By contrast, for the *healthy* group, the coefficients of PSS and SSS are also negative, but their 95% confidence intervals cross zero, suggesting that their vulnerability-mitigating effects are relatively weak for healthier rural older adult households. These findings are consistent with the predictions of resource dependence theory. Rural older adult households in poor health rely much more heavily on transfer income than healthier counterparts, so the marginal poverty-reducing effects of PSS and SSS are amplified among the unhealthy group. The coefficients on MSS are negative and statistically significant in both subsamples, and their magnitudes are very similar. These results suggest that MSS reduces vulnerability to poverty, but its marginal impact does not differ in a clear way between healthy and unhealthy older adult households. A plausible explanation is the current medical reimbursement scheme still provides limited protection in rural area, which against chronic and catastrophic illnesses among the older adults. Thus, MSS has not yet effectively blocked the risk of poverty caused by illness.

#### 4.3.3 Differences in living arrangement

Living arrangements fundamentally shape rural older adult households’ access to resources, especially informal support from adult children. To capture heterogeneity by living arrangement, we divide the sample into two groups, older adult households co-residing with their adult children and those living alone. And we re-estimate the effects of PSS, MSS and SSS on vulnerability to poverty among rural older adult households. The results are presented in Figure 5.

Panels A and C of Figure 5 show that the coefficients of PSS and SSS are significantly negative for rural older adult households living alone. This indicates that PSS and SSS constitute the key institutional safety net for these older adult households living alone and play a particularly salient role in reducing their vulnerability to poverty. By contrast, rural older adult households co-residing with adult children can largely rely on intergenerational support to share resources and pool risks, thereby reducing their marginal dependence on formal social support. Additionally, Panel B shows that the estimated coefficients of MSS do not differ significantly between the two types of living arrangement. This suggests that the current medical security does not exhibit pronounced heterogeneity in mitigating vulnerability to poverty.

### 4.4 Mechanism analysis

#### 4.4.1 Spillover effect of formal social support

Based on the theoretical analysis in the previous section, formal social support may generate spillover effects when embedded within family and community networks. These effects operate through two primary mechanisms. First, formal social support may shift intergenerational economic support toward non-economic support, such as emotional support and life caregiving, by alleviating the financial burdens of adult children. Second, formal social support may enhance neighborhood support by fostering generalized trust within community, thereby strengthening informal social support networks. Table 7 reports the results of the spillover effects. Columns (1) and (2) examine the impacts on neighborhood economic and non-economic support, and Columns (3) and (4) analyze the impacts on intergenerational economic and non-economic support. S

**Table 7:** The spillover effects of formal social support.

Variable	Informal social support			
	(1)	(2)	(3)	(4)
	NSS	NESS	CSS	CESS
PSS	0.013	0.007	0.005	0.045
	(0.018)	(0.015)	(0.008)	(0.029)
MSS	1.854^***^	−0.204	0.876^***^	0.336
	(0.241)	(0.157)	(0.116)	(0.326)
SSS	−0.011	0.011	0.011	0.073^**^
	(0.019)	(0.016)	(0.009)	(0.029)
Cons.	−26.334	2.118	−20.577	18.103
	(24.290)	(13.414)	(20.607)	(26.365)
Controls	YES	YES	YES	YES
Year FE	YES	YES	YES	YES
Household FE	YES	YES	YES	YES
Region FE	YES	YES	YES	YES
Obs.	3,258	3,258	3,258	3,258
adj. R^2^	0.131	0.005	0.114	0.024

The estimated models in Column (1), (3) and (2), (4) are OLS and Logit, respectively; Column (1) and (3) report the marginal effects; the R-squares in (1), (3) and (2), (4) columns are adjusted R-squares and pseudo-R-squares, respectively; ***, **, and * indicate significance levels at 1, 5, and 10%, respectively; Standard errors in parentheses.

As anticipated, the result in Column (1) of Table 7 shows that the coefficients for the effects of MSS on NSS is positive. This provides strong evidence of a significant spillover effect of formal social support at the community level. This finding indicates that MSS plays a pivotal role in enhancing neighborhood cohesion and promoting informal social support networks within communities. We argue that this mechanism operates by alleviating household budget constraints and reducing the need for precautionary savings. Consequently, formal social support provides the necessary material foundation for the older adults to engage in community activities and fostering generalized trust (53). These findings contribute to the academic debate on how formal and informal social support are related.

Previous studies show that this relationship is complex. For example, Chumo et al. report that although formal social support is helpful, informal networks remain essential for vulnerable groups (54). Furthermore, formal social support is often viewed as complementary to informal social support, primarily because they may lack the capacity to fully satisfy specific social and emotional needs (55). Research also suggests that when formal support is weak, informal social support becomes more important (56). In contrast, our results indicate that formal social support does not crowd out informal social support. Instead, it reinforces informal networks by enhancing the overall resilience of the community.

The results reported in Columns (3) and (4) of Table 7 indicate significant and robust spillover effects at the household level. The coefficients of MSS on CSS and SSS on CESS are both positive and statistically significant at the 1 and 5% levels, respectively. We think that MSS reduces the financial barriers to healthcare utilization and thereby releases previously suppressed healthcare demand among rural older adult households. This encourages adult children to devote more time and effort to provide companionship and caregiving for rural older adult households, giving rise to complementary care-oriented behaviors. Meanwhile, higher levels of SSS also induce adult children to provide more generous financial transfers to rural older adult households.

In summary, we identify a spillover effect of formal social support on informal social support. This spillover takes the form of a complementary relationship between formal and informal social support in reducing the vulnerability to poverty among rural older adult households. Consequently, hypothesis H2 is empirically supported.

#### 4.4.2 Regional heterogeneity of spillover effects

We further examine the heterogeneity of spillover effects of formal social support across regions, contextualized within Chinese unique patterns of population mobility driven by significant regional economic disparities. Specifically, Northeast China has experienced substantial net population outflows driven by young labor force migrating to other regions for better employment opportunities and higher incomes. To analyze this spatial heterogeneity, we construct a binary regional variable (1 = Northeast China; 0 = other regions). Furthermore, we incorporate an interaction term between formal social support and the regional variable. This approach allows us to assess the regional difference of the spillover effects of formal social support.

Table 8 reports the regional heterogeneity in the spillover effects of formal social support. The interaction terms between PSS or MSS and the regional dummy are not statistically significant in any specification. This indicates no clear difference between Northeast China and other regions in the spillover effects of PSS and MSS on NSS and CSS. In other words, the spillover effects of PSS and MSS appear broadly similar across regions. By contrast, Column (3) of Table 8 shows that the interaction term between SSS and the regional dummy is positive and statistically significant at the 10% level. This suggests that SSS generates stronger spillover effects on CSS in Northeast China than in other regions. Compared with more developed areas, rural Northeast China faces a much thinner supply of institutional and family care services. As a result, they have limited options to purchase professional care to substitute for caregiving even when older adult households receive government subsidies. Therefore, the additional resources and the sense of security brought by SSS are more likely to be locked in within the family. At the same time, large scale out migration of younger workers reduces the number of adult children who can provide care. This increases the marginal value of each return visit and period of companionship. These conditions lead to a more pronounced spillover effect of SSS in Northeast China.

**Table 8:** Regional heterogeneity of the spillover effects of formal social support.

Variable	(1)	(2)	(3)	(4)
	NSS	NESS	CSS	CESS
PSS	0.007	0.011	0.001	0.044
	(0.020)	(0.017)	(0.009)	(0.031)
MSS	1.971^***^	−0.169	0.901^***^	0.297
	(0.261)	(0.172)	(0.125)	(0.350)
SSS	−0.015	0.004	0.005	0.070^**^
	(0.020)	(0.018)	(0.010)	(0.033)
PSS × Area	0.037	−0.017	0.027	0.006
	(0.045)	(0.029)	(0.022)	(0.071)
MSS × Area	−0.980	−0.261	−0.280	0.286
	(0.684)	(0.448)	(0.361)	(0.934)
SSS × Area	0.031	0.043	0.042^*^	0.017
	(0.055)	(0.041)	(0.023)	(0.066)
Controls	YES	YES	YES	YES
Year FE	YES	YES	YES	YES
Household FE	YES	YES	YES	YES
Region FE	YES	YES	YES	YES
Cons.	−23.192	3.112	−18.200	17.066
	(25.055)	(13.605)	(20.629)	(26.499)
*Obs.*	3,258	3,258	3,258	3,258
adj. *R*^2^	0.133	0.006	0.116	0.023

***, **, and * indicate significance levels at 1, 5, and 10%, respectively; Standard errors in parentheses.

## 5 Conclusion

Our study emphasizes the critical role of formal social support in reducing the vulnerability to poverty among rural older adult households. Using CFPS data from 2018 to 2022, we show that formal social support significantly lowers the probability of falling into poverty. Under the US$1.90 per person per day poverty line, PSS, MSS and SSS reduce the likelihood of poverty by 0.314, 3.115 and 0.258 percentage points. These results confirm that formal social support helps mitigate risk shocks and stabilize the welfare of rural older adult households. These findings remain robust across alternative poverty lines, different vulnerability thresholds, and various model specifications, including conditional fixed effects logit models, a weighted panel fixed effects specification and an IV approach. Beyond its direct impact, formal social support also generates spillover effects that strengthen informal social support networks. Specifically, MSS increases trust in neighbors and strengthens non-economic support from adult children. Our results show that increases trust in neighbors and strengthens non-economic support from adult children, while SSS is associated with higher financial transfers from adult children. These mechanisms suggest that when basic income and health risks are buffered by formal programs, adult children can shift part of their support from subsistence to care and emotional companionship, and older adult households can more actively participate in community interactions. These findings are consistent with international evidence documenting that formal social protection often coexists with informal care and social capital rather than simply displacing it.

Our study also underscores pronounced heterogeneity across regions, health condition and living arrangements. From a regional perspective, PSS is most effective in the central and western provinces, where rural older adult households have weaker capacity to respond to risk. MSS shows the strongest poverty reducing effect in the more developed eastern region, while SSS is particularly important in the central and western regions. These reflect long standing regional gaps in economic development and in the supply of social programs. They suggest that region specific strategies are needed rather than a uniform expansion of social programs across all areas. From a health perspective, we find that PSS and SSS are most effective for older adult households in poor health, which is consistent with the predictions of resource dependence theory. By contrast, MSS in rural areas still struggles to address the cumulative risks and costs associated with chronic conditions and major illnesses. This indicates that strengthening the development of the medical security system is important in China’s rural areas. From a living arrangements perspective, we find that PSS and SSS are especially important for older adult households who live alone. These effectively activate informal social support at the community level, providing the emotional support that the older adult households require. Overall, these heterogeneous results reinforce the core claim of our risk shock-response framework, which formal social support is more effective for groups that are structurally more vulnerable.

Based on these findings, we propose the following policy recommendations. Firstly, medical and pension security systems should be improved. Because older adult households face high health risks and have limited capacity to respond to shocks, health insurance design should include service packages for chronic disease management that target low-income older adult households. These services can help prevent a cycle in which health shocks lead to further welfare decline. At the same time, pension security schemes should adopt age graded benefit levels so that welfare standards are higher for the oldest age groups.

Secondly, the government should create incentives to activate informal social support. Given the positive spillover effects of formal social support, at the household level, fiscal incentives can be used to reduce the financial burden on adult children. For example, adult children who provide regular care to the older adult households can receive modest care allowances. At the community level, the government can purchase services to supply the non-economic support that older adult households need, such as community service centers for the older adults, public canteens and spaces for social activities. These measures can help raise interpersonal trust within communities and foster more sustainable informal support networks.

Finally, given the regional differences in the poverty reducing effect of SSS, the government should provide higher levels of government assistance to regions with large population outflows. Assistance levels can be linked to local migration rates and to the number of older adult households whose adult children work away from home, so that the older adults who lack informal social support receive basic security. Policy design should also take group differences into account. Social security for the oldest and for the older adults who live alone should be strengthened, with particular attention to residents in high migration regions. Policy measures may include raising minimum benefit standards, subsidizing insurance premiums and expanding the supply of community-based care facilities.

## Data Availability

The data analyzed in this study is subject to the following licenses/restrictions: the datasets used in this study (CFPS) are available from the official CFPS website upon successful application. In accordance with CFPS policy, these datasets are distributable only by the CFPS team. Requests to access these datasets should be directed to https://www.isss.pku.edu.cn/cfps/.

## Footnotes

1 Data source: https://www.who.int/zh/news-room/fact-sheets/detail/ageing-and-health.

2 Data source: https://www.stats.gov.cn/xxgk/jd/sjjd2020/202401/t20240118_1946711.html.

3 Data source: http://finance.people.com.cn/GB/n1/2016/0707/c1004-28531389.html.

4 Data source: https://www.gov.cn/guoqing/2021-05/13/content_5606149.htm.

5
https://sdg.iisd.org/news/world-social-report-2023-calls-for-leaving-no-ageing-person-behind

6 https://www.who.int/health-topics/social-determinants-of-health#tab=tab_1.
